# Carbon dots as photosensitizers: unraveling their ultrafast charge transfer, challenges, and future prospects

**DOI:** 10.1039/d5na00806a

**Published:** 2025-12-08

**Authors:** Somen Mondal

**Affiliations:** a Institute of Chemical Technology, Mumbai Marathwada Campus Jalna Maharashtra 431203 India somen.md@gmail.com

## Abstract

Carbon dots (C-dots) have emerged as highly promising light-harvesting materials, particularly as photosensitizers, due to their eco-friendliness, biocompatibility, and cost-efficiency. Their adaptability as photosensitizers has garnered widespread attention, marking them as pivotal materials for future technological innovations. One of the key attributes of C-dots is their dual functionality in charge transfer, enabling them to serve as both electron donors and acceptors. This charge transfer process between C-dots and small organic molecules as quenchers plays a critical role in diverse applications such as photocatalysis, sensing, and optoelectronics. In this perspective, we have discussed the capability of C-dots in confined environments, doped C-dots, C-dots/molecular hybrids, and perovskite/C-dots composites as photosensitizers. This perspective includes the origin of fluorescence and carrier dynamics in full colour light-emitting C-dots, followed by a novel way to control the photosensitizer capability of C-dots *via* the transfer of electrons and holes in hybrids, composites, and doped C-dots, and the effects of the core and surface in the electron transfer process. The photosensitizer capability of C-dots was investigated *via* exploring the charge transfer dynamics using various advanced optical techniques like steady-state and time-resolved photoluminescence and ultrafast transient absorption (TA). This perspective also focuses on understanding the ultrafast dynamics of C-dots, such as charge transfer, charge transport, and charge recombination, in various environments, composites, and hybrid systems, with an emphasis on their development as effective photosensitizers. The extensive range of reported electron donor–acceptor systems underscores the versatility of C-dots as photosensitizers, with their tuneable electronic properties tailored to address the demands of emerging technological challenges.

## Abstract 2

Versatility of C-dots as photosensitizers in confined environments including doped C-dots, C-dot/molecular hybrids, and perovskite–C-dot composites.

## Graphical abstract

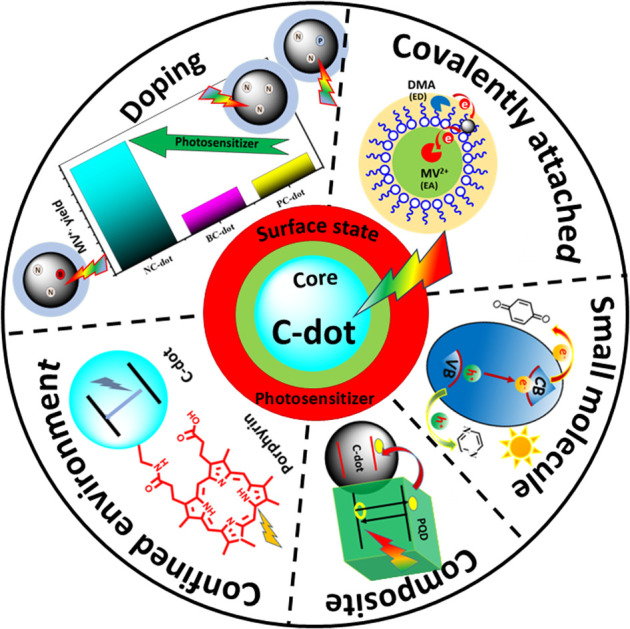

## 1. Introduction

Carbon dots (C-dots), zero-dimensional allotropes of carbon, are one of the most exciting nanomaterials since their inception due to their mysterious nature. C-dots were first discovered as by-products during the purification of single-walled carbon nanotubes (SWCNTs) using gel electrophoresis in 2006.^1–4^ Since then, C-dots are often considered as small carbon nanoparticle composites of a core state along with surface functional groups in the carbon family.^5–10^ C-dots gained significant traction in the last decade among the scientific community due to their easy functionalization, high aqueous solubility, low toxicity, high quantum yield, high photostability, and chemical inertness.^6,11–13^ Accordingly, an increasing number of research publications on C-dots can be observed in areas such as optoelectronics, photocatalysis, energy storage, drug delivery, bioimaging, and sensing.^5,8,14–20^ Over the past decade, numerous scientific research groups have dedicated their efforts to fully understand the structure and optical properties of C-dots.^12,13,17,18,21–23^ The vast variety of structural features and optical properties, depending on the starting materials, has led to unique materials such as “carbon-based nanoparticles”, “carbon dots”, “carbon quantum dots”, and “carbogenic dots”.^24,25^ Due to their complexity and diversity in photophysical behaviours, two widely accepted perspectives have emerged regarding the origin of photoluminescence (PL) and the heterogeneous nature of C-dots. The most widely accepted explanation suggests that C-dots contain two distinct emissive species. Blue emission originates from the core state, attributed to an sp^2^-hybridized aromatic network surrounded by an amorphous sp^3^-hybridized domain. In contrast, green and red emissions arise from the surface state, influenced by functional groups located at the edges of the carbon core.^11,23,26–28^ An alternative perspective proposes that the emissions result from the formation of molecular fluorophores or aggregated structures within the carbon core during synthesis.^29–34^

C-dots can be synthesised *via* two main synthetic approaches: top-down and bottom-up.^35^ Properties of C-dots are not solely determined by their chemical components but are also heavily influenced by their synthesis conditions.^31,36–38^ The electrochemical or chemical oxidation of graphite can yield relatively large quantities of C-dots in top-down methods. Moreover, graphene oxide,^39^ coal,^40^ CNTs,^41^ carbon black, candle soot,^42^ and activated carbon^43^ have been extensively used to synthesize C-dots *via* chemical oxidation (acidic), surface passivation, hydrothermal synthesis and microwave-assisted cutting. In the bottom-up approach, small organic molecules are used as the sources of C-dots, and pyrolysis or carbonization^44–46^ and hydrothermal,^47,48^ solvothermal,^49,50^ and microwave-assisted methods^51,52^ are applied for the successful synthesis of C-dots. Bottom-up approaches provide greater versatility in C-dot synthesis due to enhanced control over structure and functionality.^35^ Moreover, this method is flexible in terms of reaction conditions such as thermal treatments and reaction times, with the advantages of low cost and environmental friendliness.^31^ Citric acid, glucose and amino acids, along with some amine-containing surface coating agents, have been used widely for the synthesis of C-dots in bottom-up synthesis methods.^35^ The versatility regarding the choice of precursors and the methods of synthesis affects the chemical properties, optical properties, size, graphitization degree in the core, surface functional groups of the surface state, and heteroatom doping in the core.^10,14,15^ Thus, the uniform and reproducible synthesis of C-dots is challenging for researchers. Isolating the products from a mixture of C-dots also plays an important role in controlling the optical and surface properties of C-dots. Dialysis is the most used purification method for C-dots reported in the literature, but only the small capping ligands or ions can be removed from the mixture. Thus, blue-emitting C-dots (B-C-dots), green-emitting C-dots (G-C-dots), and red-emitting C-dots (R-C-dots) can be separated by high-separation-efficiency techniques, such as high-performance liquid chromatography, column chromatography, or gel electrophoresis.^10,23,53^ Thus, researchers are using machine learning as a tool for predicting the material descriptors and desired properties to overcome the challenges in optimizing the synthesis of C-dots.^15,54–56^

**Scheme 1:**
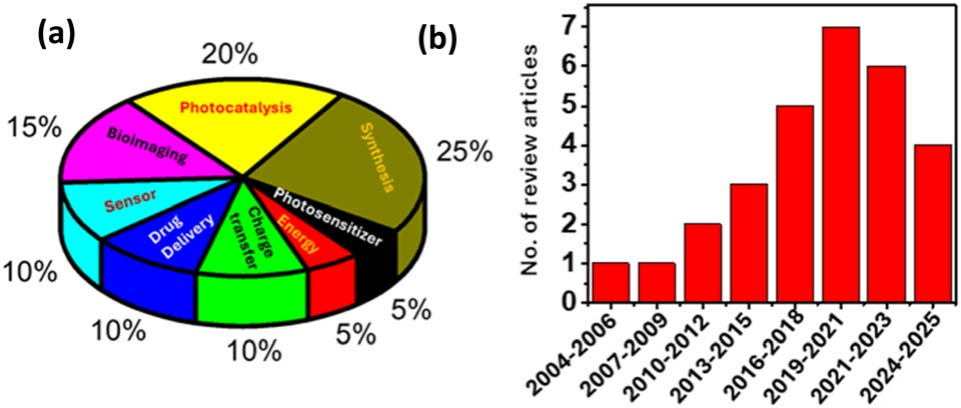
(a) Pie chart showing the applications of C-dots. (b) Number of perspective articles published on charge transfer processes in C-dots from 2004 to 2024; search results were obtained from AI-based statistics.

Artificial intelligence (AI)-based statistics show that the majority of perspective articles cover the fields of synthesis, photocatalysis, bioimaging, drug delivery, sensor, and charge-transfer processes; however, C-dots as photosensitizers, and the associated charge transfer and separation processes in C-dot-quencher systems, have not been covered in many articles.^5–8,12–15,17–20,57,58^ To investigate the quenching photoexcited electron transfer process (PET), various electron acceptors (EAs), such as 4-nitrotoluene,^59^ 2,2,6,6-tetramethylpiperidine (TEMPO),^60^ methyl viologen (MV^2+^),^26,27,61,62^ metal nanoparticles,^63^ fumarate reductase, [NiFeSe]-hydrogenase,^64,65^ and metal complexes^66,67^ have been employed to explore the electron-accepting properties of C-dots. Similarly, C-dots have been used in PET processes with different electron donors (EDs) like *N*,*N*-diethylaniline (DMA),^62^ triethanolamine (TEOA),^68^ porphyrins,^69–73^ and ethylenediaminetetraacetic acid (EDTA).^68^ Therefore, this account addresses the charge-transfer process in full-colour light-emitting C-dots, C-dots in confined environments, doped C-dots, C-dots/molecular hybrids and perovskite/C-dots composites upon the photoexcitation of C-dots, based on research conducted in our laboratory over the past five years.^11,23,26,27,61,62,74–77^ Additionally, we explore how the donor–acceptor properties of C-dots conjugated with dopamine can be tuned by varying the pH of the medium, as well as how aggregation-induced electron transfer occurs in C-dot–MV^2+^ composite systems.^61,78^ To provide proper context for our work on tuning charge transfer in C-dots, we will begin by outlining our efforts to investigate the electronic structure of prototype C-dots. We will then situate our findings within the broader landscape of current research on C-dots, particularly in charge-transfer applications, which extend into various fields such as photocatalysis,^79^ optoelectronics,^80,81^ bioelectronics,^82^ bioimaging,^83^ sensing,^84,85^*etc.* A pie chart is provided to illustrate the versatility of C-dots in various applications, including photocatalysis, energy storage, drug delivery, bioimaging, sensing, and charge transfer processes, as shown in Scheme 1a. The number of perspective articles published on charge transfer processes involving C-dots from 2004 to 2024 has steadily increased, as depicted in Scheme 1b; however, the number of perspectives in this area remains lower compared to other application fields.

An ultrafast spectroscopic study revealed that the origin of green fluorescence in both C-dots and graphene quantum dots arises from similar molecule-like emissive states associated with carboxyl and carbonyl functional groups.^28^ Feldmann and co-workers conducted the ultrafast TA of C-dots in the pure acetonitrile solution and observed only a broad and structureless excited state absorption (ESA) in the entire probe region caused by the photoexcited carriers. A stimulated emission (SE) band is observed in the photoexcited C-dots upon the addition of water in the acetonitrile solution, indicating the proton affinity of C-dots due to its photobasic effect.^86^ Ghosh *et al.* reported the ultrafast carrier dynamics of red-emitting C-dots and boron-doped red-emitting C-dots, where the introduction of boron led to the formation of an additional excited-state level in C-dots. This newly formed state captured a significant portion of the excited-state population, thereby slowing down the initial relaxation process toward the emissive state.^87^ Recently, ultrafast TA spectroscopy revealed that thiol-functionalized C-dots facilitate rapid and efficient hole transfer from photoexcited C-dots to the thiol groups, initiating the formation of boryl radicals through a cooperative hydrogen atom transfer process. This mechanism significantly enhances the photocatalytic activity of C-dots.^88^

In this perspective, we discuss the major advancements made in understanding the origin of carbon dots (C-dots) by exploring their ultrafast carrier dynamics across differently emitting C-dots—an area that has received limited attention in the C-dot research community. The charge transfer and recombination processes in confined environments, doped C-dots, C-dot/molecular hybrids, and perovskite/C-dot composite systems are examined to provide direct evidence of their photosensitizing capabilities. Furthermore, the significant influence of both the core and surface states of C-dots on the electron transfer process is highlighted. Thus, this perspective highlights recent advancements from our laboratory on the ultrafast charge transfer processes in C-dots investigated over the past few years and seeks to bridge the knowledge gap concerning the charge-transfer mechanisms triggered upon photoexcitation of C-dots.

## 2. Photophysical properties and carrier dynamics of C-dots

**Fig. 1:**
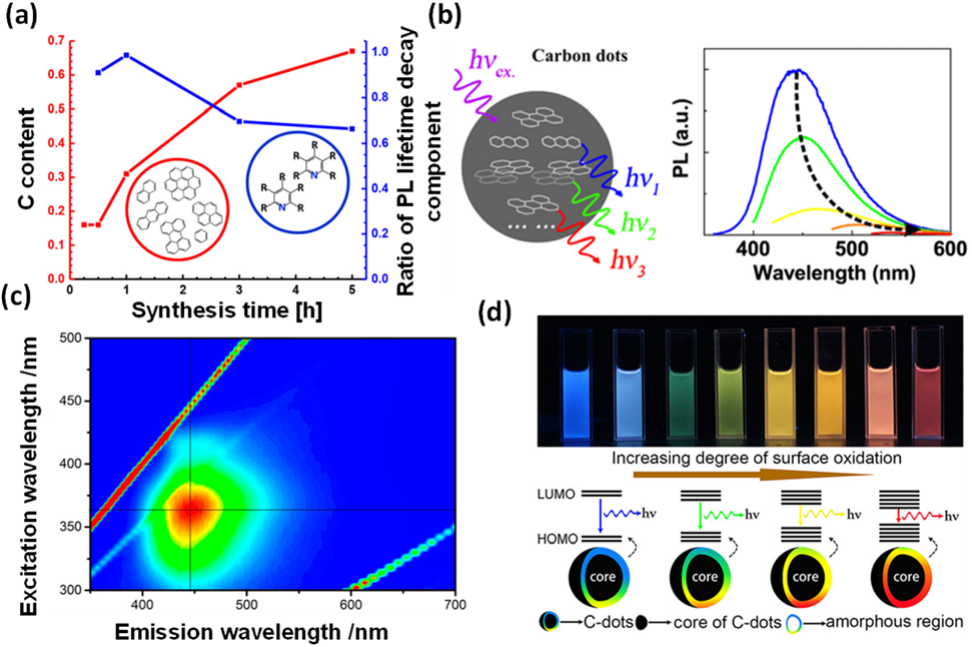
(a) Carbon content in C-dots (red line) increases with the progress of the reaction and the ratio (blue line) between the long and short decay components of the fluorescence lifetime, adapted from ref. 29 with permission from the American Chemical Society, copyright 2025. (b) Schematic depicting the formation of polycyclic aromatic hydrocarbon molecules as the origin of PL, adapted from ref. 30 with permission from the American Chemical Society, copyright 2025. (c) PL of C-dots in a 3D plot, where the intensity increases from blue to green and to red, adapted from ref. 68 with permission from the American Chemical Society, copyright 2025. (d) Images of full-colour light-emitting C-dots under a 365 nm UV light; the band gap can be tuned by increasing the surface oxidation, adapted from ref. 89 with permission from the American Chemical Society, copyright 2025.

The photophysical properties of C-dots are challenging to investigate due to their complex structures and the lack of proper purification techniques. Therefore, the isolation of different emitting C-dots from a mixture using appropriate purification methods is essential to exploring the origin of their fluorescence and excitation-dependent emission properties. To investigate the photophysical properties of C-dots, Ehrat *et al.* synthesized C-dots using citric acid and ethylenediamine under varying reaction times in a hydrothermal process. Their study revealed that molecular fluorophores form during the initial phase, while aromatic domains develop and grow as the reaction progresses, with no significant change in the overall particle size (Fig. 1a). The formation of these aromatic domains was found to induce a rapid increase in PL emission and enhance the PL quantum yield (QY) of C-dots.^29^ Fu *et al.* reported the formation of three types of polycyclic aromatic hydrocarbon molecules responsible for the PL of C-dots. They also suggested that the observed Stokes shift in C-dots arises from exciton self-trapping within the network, as shown in Fig. 1b.^30^ The 3D luminescence plot (Fig. 1c) exhibited narrow, excitation-independent PL characteristics, indicating that the fluorescence originates from a single particle species.^68^ Ding *et al.* synthesized blue-, green-, yellow-, and red-emitting C-dots with emission maxima ranging from 440 to 625 nm, as shown in Fig. 1d. They found that the energy band structures were governed by the surface groups and chemical structures rather than the particle size.^89^

Dialysis is the most commonly used method for the purification of C-dots reported in the literature. However, dialysis is effective for removing ions and small capping ligands from a mixture of differently emitting C-dots, it is not suitable for separating specific emitting C-dots such as B-C-dots, G-C-dots, and R-C-dots. To address this limitation, we synthesised B-C-dots, G-C-dots, and R-C-dots from a single carbon source by dissolving citric acid and urea, and separated them using column chromatography. The origin of electronic transitions was verified using absorption studies, where B-C-dots, G-C-dots, and R-C-dots displayed absorption maxima at 340, 420, and 540 nm. These transitions originated from the core and the surface (oxygen-containing functional groups) of the particles. Furthermore, all C-dots exhibited emission peak maxima at ∼440 nm, ∼520 nm, and ∼610 nm when excited at 340 nm, 420 nm, and 500 nm, respectively. This indicates that the 440 nm emission is an inherent property of all C-dots, arising from transitions in the core state. The green and red emission peaks, however, originate from the surface state of C-dots, where functional groups are attached to the edges of the carbon core.

**Fig. 2:**
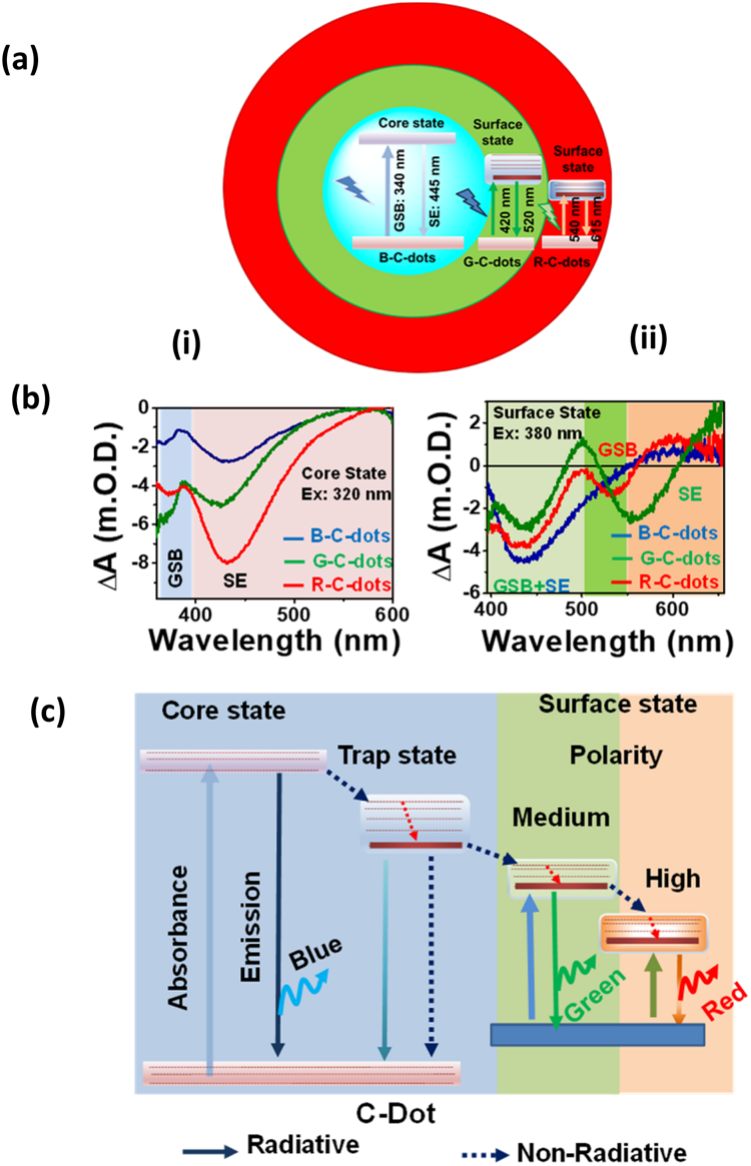
(a) Representation of the different emissive states and radiative processes in C-dots. (b) TA spectra of the (i) core state upon excitation 320 nm and (ii) surface state after a 380 nm excitation. (c) Schematic of different relaxation pathways for blue-, green- and red-emitting C-dots, adapted from ref. 23 with permission from the Royal Society of Chemistry, copyright 2025.

To investigate the carrier dynamics and the origin of emissive centres in differently emitting C-dots, we performed time-resolved PL and TA measurements after selectively exciting the core and surface states of C-dots. Time-resolved PL measurements were carried out by exciting all the emitting C-dots at 340 nm and 405 nm while monitoring emissions at 440 nm and 530 nm as shown in Fig. 2a. The decay kinetics of the core state, monitored at 440 nm following excitation at 340 nm and 405 nm (corresponding to the core state's band edge), revealed similar carrier-relaxation pathways for all C-dots. In contrast, the decay kinetics of the surface state showed a growth component in the R-C and G-C-dots, attributed to solvent relaxation around the surface functional groups near carbon-like peripheral structures. This growth component was absent in the B-C-dots, likely due to the lower solvation capability of their surface functional groups. The most widely accepted explanation for the fluorescence origin is that the core, present in all emitting C-dots, serves as the centre of blue emission. Meanwhile, surface states containing various oxygen-functional groups undergo solvation, modulating green and red emission maxima by creating distinct fluorescence centres in C-dots.^10,23,28,68,90,91^

Ultrafast TA measurement was carried out to follow the radiative and non-radiative processes in different emitting C-dots and how the charge-transfer state is populated through the selective excitation of the core and surface states at 320 and 380 nm, respectively, and then probing the output signal in the UV-vis range, as shown in Fig. 2b(i) and (ii). All C-dots exhibited similar spectral features at 360 nm for ground state bleaching (GSB, negative signal) and 430 nm for stimulated emission (SE, negative signal) when excited at 320 nm, suggesting the presence of comparable energy levels at the cores of the various emitting C-dots as shown in Fig. 2b(i). However, two negative absorption bands at 400–500 nm (GSB of the surface state and SE of the core state) and 540 nm (SE) were observed upon the excitation of the surface states of the differently emitting C-dots at 380 nm, as shown in Fig. 2b(ii). The presence of two GSB bands clearly indicated the existence of two states, namely, the core state (340 nm) and surface state (420 nm), in all C-dots, supporting the occurrence of fluorescence in the blue and green regions from the two different states. Moreover, R-C-dots showed GSB and SE at 520 nm and a broad peak at 540–620 nm, respectively, after excitation at 500 nm, which was responsible for the red emission from C-dots. The findings indicate that the highly oxygen-rich surface functional groups are responsible for the red emission in C-dots. Polarity-dependent carrier-relaxation pathways from the different excited states of the emitting C-dots are shown in Fig. 2c. The fluorescence and excitation-dependent emissions originate from the core and different surface states, where the core is an intrinsic property of all emissive C-dots.^23^

## 3. Effects of the core and surface states in the ET process in different emitting C-dots

After successfully establishing the presence of core and surface states in all emitting C-dots and exploring the carrier dynamics of the core and surface states, herein, we discuss the role of heterogeneity in the core state and surface state of C-dots in the ET process. The ET process between different emitting C-dots and an electron acceptor methyl viologen (MV^2+^) was investigated using spectroscopic techniques to determine the roles of core and surface state.

**Fig. 3:**
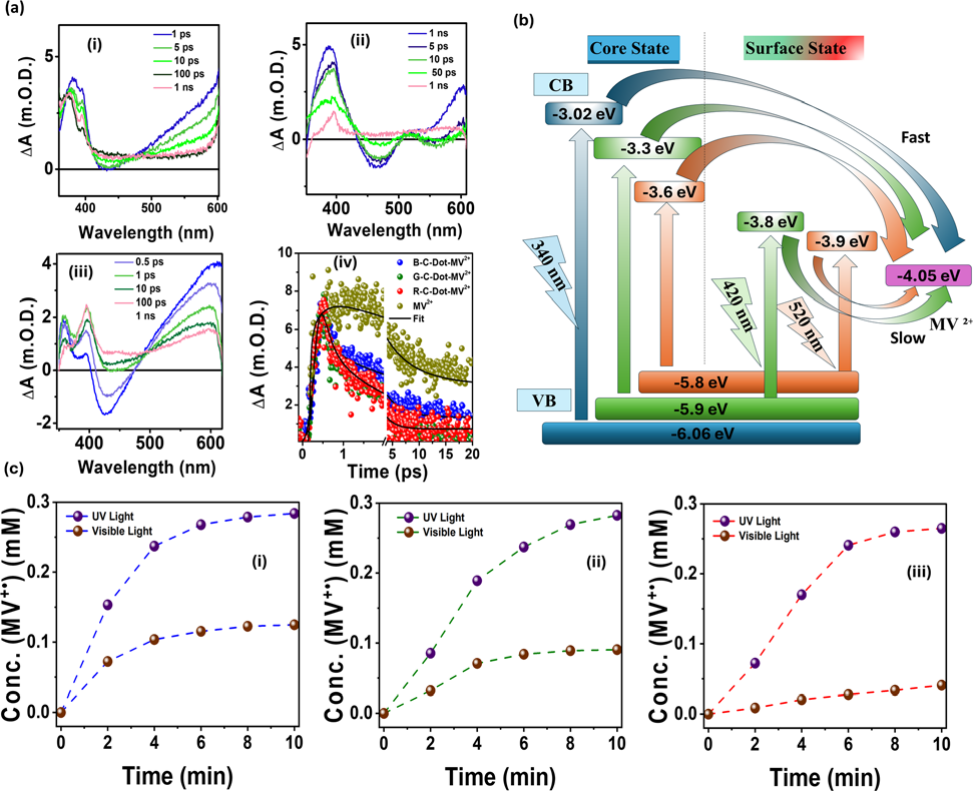
(a) TA data for (i) B-C-dots, (ii) G-C-dots, and (iii) R-C-dots at different time delays, and (iv) kinetics of MV^2+^ and all C-dot–MV^2+^ composites, probed at 600 nm after a 320 nm excitation. (b) ET pathways of different emitting C-dots in the presence of MV^2+^ after the selective excitation of the core and surface states. (c) Time-dependent concentrations of MV^+^˙ after irradiation with UV and visible lights for (i) B-C-dots, (ii) G-C-dots, and (iii) R-C-dots in the presence of the sacrificial electron donor triethanolamine (TEOA). Adapted from ref. 26 with permission from the Royal Society of Chemistry, copyright 2025.

The steady-state and time-resolved PL show the different extent of quenching for the ET process in the core state and surface state of C-dots due to structural heterogeneity. Zbořil and co-workers demonstrated that graphitic nitrogen introduces midgap states within the HOMO–LUMO gap of C-dots. This occurs due to the donation of excess electrons into the unoccupied π* orbitals, which narrows the HOMO–LUMO gap and leads to a red-shifted emission in C-dots. Furthermore, nitrogen doping contributes additional electrons to the π* orbitals of the conjugated system.^92^ Our experimental study revealed that the core domain of C-dots generates an energy level (N-state) associated with nitrogen atoms, which facilitates the ET process in R-C-dots, G-C-dots, and B-C-dots. This transformation renders the core an effective electron donor, in contrast to the surface state, which is dominated by oxygen-rich functional groups that act as strong electron traps. TA spectra (Fig. 3a(i)–(iii)) showed the complete disappearance of the GSB band and the appearance of a positive excited state absorption (ESA) band at 600 nm, indicating the formation of MV^+^˙ upon excitation of the core state, but no signal was observed at 600 nm after the excitation of the surface state. Kinetics data showed that the ET process from photoexcited C-dots to MV^2+^ is faster from the core state as compared to the surface state, as shown in Fig. 3a(iv). This indicates the existence of heterogenicity in the core and surface states during the ET process, and the core state acts as a better photosensitizer than the surface state. The ET process after excitation of the core and surface of C-dots was described in Fig. 3b. The yield of MV^+^˙ generated in the core state was 0.3 mM for all emitting C-dots, but yields of 0.12, 0.09, and 0.04 mM were obtained for the surface states of B-C-dots, G-C-dots, and R-C-dots, respectively, calculated from Fig. 3c(i)–(iii). In summary, the higher PET efficiency observed in the core state of all emitting C-dots makes them superior photosensitizers compared to those dominated by surface-state processes.^26^ After investigating the ultrafast dynamics of C-dots, a summary of the photoluminescence origin, carrier cooling, and charge recombination times for blue-, green-, and red-emitting C-dots is presented in Table 1.

**Table 1:** Summary of charge transfer and recombination processes (*τ*_dir._ = direct recombination, *τ*_trap_ = trap-mediated recombination, *τ*_rad._ = radiative pathway, *τ*_non-rad._ = non-radiative pathway), including the origin of photoluminescence (PL) and the excitation and emission maxima of blue-, green-, and red-emitting C-dots^23,26^

	Carrier cooling		Charge recombination				*λ*_ex_ (nm)	*λ*_em_ (nm)	Origin of PL
	*τ*_core_ (fs)	*τ*_surface_ (fs)	Core		Surface				
			*τ*_dir._ (ns)	*τ*_trap_ (ns)	*τ*_rad._ (ns)	*τ*_non-rad._ (ps)			
B-C-dots	<100, 250	230	1.5	9.5	8.3	3.6	340	445	Core state
G-C-dots	<100, 550	170	1.8	10	8.3	5.6	420	520	Medium polar amine/oxygen-containing surface state
R-C-dots	<100, 1100	120	2	10.2	8.3, 1.07	5.7, 10.5	540	615	Highly polar oxygen-enriched surface state

## 4. Photosensitizer capability in confined environments

**Fig. 4:**
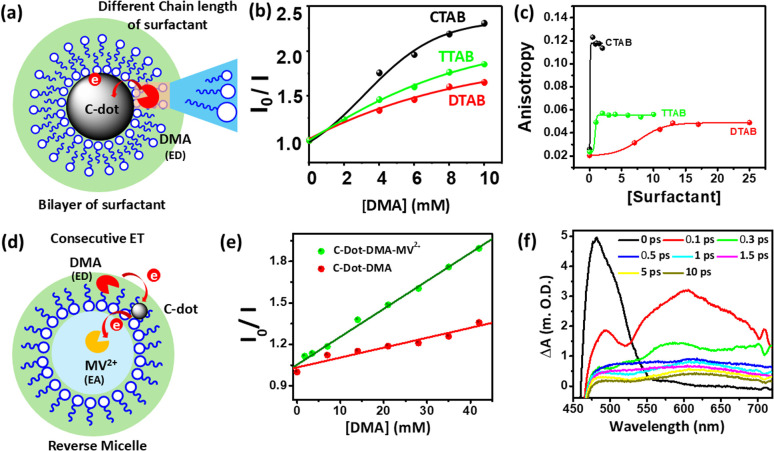
(a) Surfactant chain length controls the ET process in cationic surfactant (CTAB, TTAB, and DTAB) bilayer-protected C-dots. (b) Extent of C-dot quenching by DMA as a function of the chain length of the surfactant coated around C-dots in aqueous medium. (c) Steady state anisotropy of C-dots upon interaction with cationic surfactants with different chain lengths; adapted from ref. 62 with permission from the American Chemical Society, copyright 2025. (d) Successive ET processes in encapsulated C-dots and DMA (donor, in a nonpolar environment) and MV^2+^ (acceptor, in a polar environment). (e) Relative quenching of C-dots by DMA in the presence and absence of MV^2+^. (f) TA spectra of DMA–CNP–MV^2+^ in AOT/hexane reverse micelles following a 400 nm laser pulse excitation; adapted from ref. 75 with permission from the American Chemical Society, copyright 2025.

A confined environment refers to a restricted space where the physical and chemical properties differ significantly from those observed in bulk systems. Examples include nanopores, micelles, reverse micelles, liposomes, and polymer matrices, which provide well-defined microenvironments capable of influencing and controlling the PET process *via* restricting the motion of solvent molecules and enhancing the ET efficiency by introducing proximity effects, reducing degrees of freedom, and strengthening molecular interactions. To explore their photosensitization capabilities, C-dots and an electron donor, namely, dimethyl aniline (DMA), were incorporated into micelles (Fig. 4a), where surfactant-protected core–shell C-dots (bilayer) were developed using cationic surfactants with varying chain lengths: cetyltrimethylammonium bromide (CTAB), dodecyltrimethylammonium bromide (DTAB), and tetradecyltrimethylammonium bromide (TTAB). In Fig. 4b, it was observed that PET efficiency increased with the expansion of the shell width, as longer surfactant chains led to higher molecular compactness, thereby reducing solvent reorganization energy. The effect of chain length on molecular compactness was investigated using steady-state anisotropy measurements, as shown in Fig. 4c. Additionally, the role of C-dots as efficient photosensitizers was explored within the confined environment of reverse micelles. Here, DMA was incorporated into the nonpolar region, while MV^2+^ was positioned in the polar environment, as shown in Fig. 4d. The synergistic influence was observed in the ET process when both electron acceptor and donor were confined in reverse micelles, facilitating a consecutive ET process from DMA to MV^2+^*via* C-dots, as illustrated in Fig. 4e. The consecutive ET processes were investigated *via* fs-TA spectroscopy, where an ESA band was observed at 470 nm, along with a peak at 600 nm, confirming the formation of the DMA radical with MV˙^+^ cation, as shown in Fig. 4f. This indicates that the photosensitizing capability of C-dots can be controlled by their confined environments. The efficient PET within confined environments enables C-dots to function as effective photosensitizers, providing valuable insights for their applications in photocatalysis and light-driven chemical reactions.^62,75^

## 5. Exploitation of the photosensitizer capability of C-dots *via* doping

**Fig. 5:**
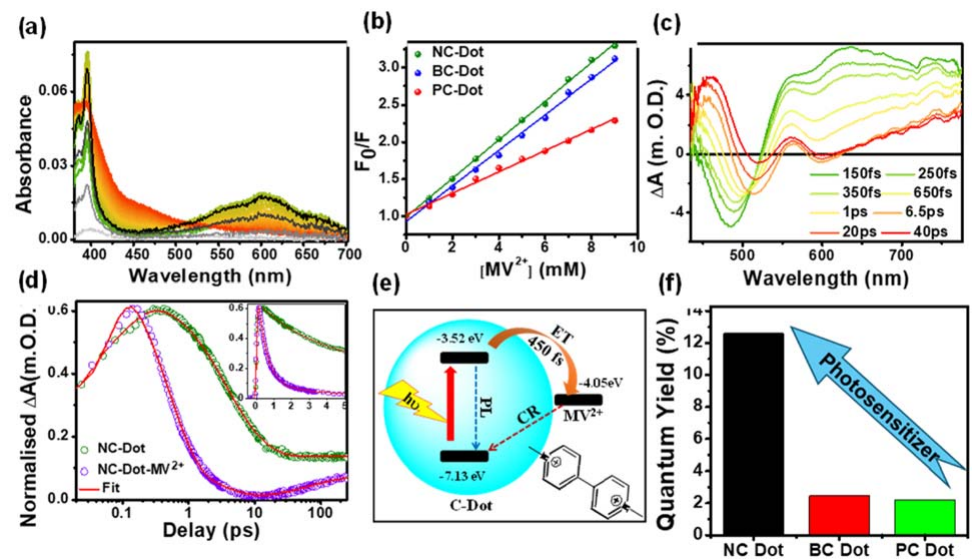
(a) Investigation of MV˙^+^ radical generation in mixtures of NC-dots with MV^2+^ systems after irradiation with light at 340 nm at different times (0–10 min in 30 s intervals) using steady-state absorbance spectra. (b) Stern–Volmer relative fluorescence quenching plots for the different doped C-dots. (c) TA spectra of NC-dots/MV^2+^ systems after excitation with a 320 nm laser pulse. (d) TA decay kinetics of NC-dots at 630 nm in the absence and presence of MV^2+^ (the inset shows a zoomed-in view of the first 5 ns). (e) Schematic of the various CT processes taking place in the doped C-dots/MV^2+^. (f) Photosensitizing ability of C-dots, which is dependent on the doping of heteroatoms in the core state due to different extents of charge delocalization. Adapted from ref. 27 with permission from the American Chemical Society, copyright 2025.

The photosensitizer capability of C-dots was investigated after doping with various heteroatoms such as nitrogen, phosphorus, sulphur, and boron in the core state of C-dots. This perspective discusses the carrier dynamics of three doped C-dots, where boron and phosphorus are doped with N-doped C-dots (NC-dots, BC-dots, and PC-dots) to investigate the effect of doping on the photosensation capability of C-dots. UV-vis spectroscopy (Fig. 5a) showed a distinct absorption band at 605 nm, confirming the formation of MV^+^˙. The Stern–Volmer plot, as shown in Fig. 5b, confirms that the extent of quenching is greater for NC-dots than for BC- and PC-dots, which implies that the NC-dots act as electron donors. Further, fs-TA spectroscopy of the different doped C-dot–MV^2+^ composite (Fig. 5c) showed a broad positive ESA band at 550–750 nm (*λ*_peak_ = 630 nm), revealing the formation of MV^+^˙. The decay kinetics at 630 nm (Fig. 5d) show that the ET is faster in the doped C-dot–MV^2+^ composite as compared to C-dots. Moreover, NC-dots exhibited slower charge recombination (CR) rates than BC- and PC-dots. These results suggest that the photoexcited C-dots transfer the electrons to MV^2+^ (−4.05 eV) due to proper band alignment, as shown in Fig. 5e with ET time. Finally, the photosensitizing performance of the different C-dots was assessed by calculating the quantum yield of MV^2+^ radical generation, as depicted in Fig. 5f. The NC-dots exhibit a higher radical generation quantum yield trend than BC- and PC-dots, which correlates well with the ET time from excited C-dots to MV^2+^ and average CR rates. These results indicate that the PET process can be effectively tuned through heteroatom doping in C-dots. The faster ET and slower CR processes in N-C-dots make them promising candidates for photosensitizer applications, supporting the design and development of efficient C-dot-based materials for heterogeneous catalysis.^27^

## 6. C-dots as a photosensitizer in C-dots/molecular hybrid

The fundamental charge transfer processes at the interface of C-dots and molecular quenchers are important for the development of novel photocatalysis, dye-sensitized solar cells and molecular electronics. This perspective summarizes the understanding of the charge transfer dynamics between C-dots and molecular composites *via* non-covalent and covalent bonding. Steady-state and time-resolved PL and fs-TA were used to investigate the ultrafast charge transfer process at the C-dot-molecule interface to explore the mechanism of photocatalysis and the long-range charge transfer process.

## 7. C-dot small organic molecule composite

**Fig. 6:**
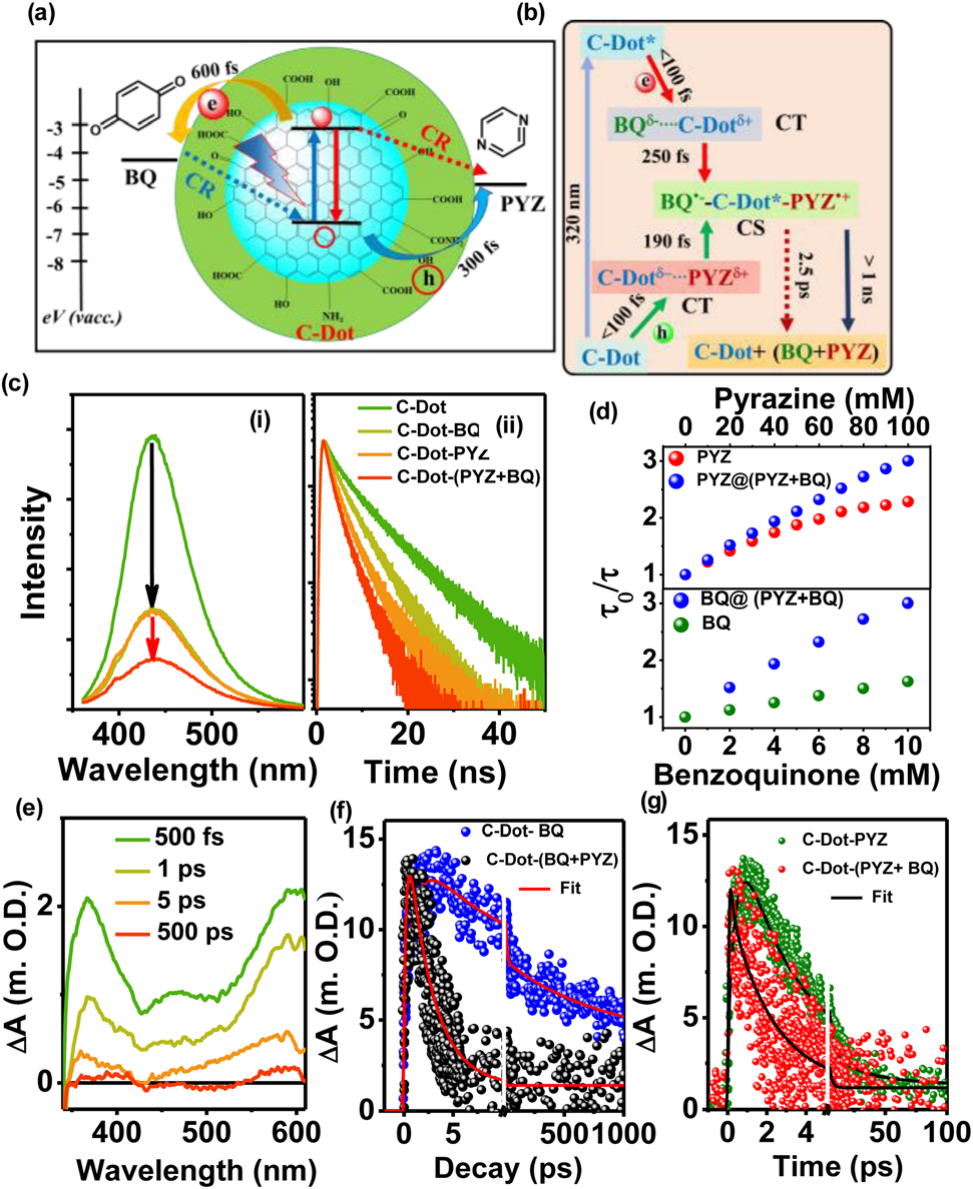
(a) Different ET, HT and charge recombination pathways between C-dots and pollutants upon light irradiation on C-dots. (b) Diagram illustrating the different deactivation pathways of the C-dot–(BQ + PYZ) system, along with their corresponding time. Quenching study of C-dots with individual quenchers (BQ and PYZ) and their mixture (PYZ + BQ): (c) (i) steady state fluorescence and (ii) time-resolved PL. (d) Extent of quenching: (top) C-dot–(PYZ + BQ) and C-dot–PYZ and (bottom) C-dot–(PYZ + BQ) and C-dot–BQ. (e) TA spectra of C-dot–(PYZ + BQ) after excitation at 320 nm. (f) Kinetics of the formation of anionic BQ at 400 nm and (g) cationic PYZ at 500 nm in the presence of individual quenchers and their mixture. Adapted from ref. 77 with permission from the American Chemical Society, copyright 2025.

This section focuses on some noncovalently interacting systems between C-dots and small organic molecules. In this work, we used C-dots as photosensitisers with some water-soluble pollutants, such as benzoquinone (BQ) acting as a sacrificial electron acceptor and pyrazine (PYZ) acting as a sacrificial hole acceptor. In this perspective, we focus on the interfacial charge separation and recombination processes between C-dots and electron acceptor BQ/electron donor PYZ or in the mixture of BQ and PYZ to determine the exact mechanism for efficient homogeneous photocatalysis due to the proper alignment of the redox energy level with the photosensitizer C-dots, as shown in Fig. 6a and b. The PL study (Fig. 6c(i)) showed almost 64% quenching with the individual addition of BQ and PYZ, but almost 80% quenching was observed after the addition of a PYZ + BQ mixture, due to simultaneous ET and HT from the photoexcited C-dots. In Fig. 6c(ii), the PL decay of C-dots became faster with the addition of BQ and PYZ, but a significant reduction in the lifetime was observed upon the addition of mixtures of BQ and PYZ. This suggests that a simultaneous electron and hole transfer process slows down the recombination process in the C-dot-mixture system. The mixture (PYZ + BQ) showed a greater extent of quenching than the individuals, as shown in Fig. 6d, indicating that the extent of charge separation is much more efficient when both ET and HT are present with C-dots. The fs-TA spectra of C-dots and the mixture of PYZ + BQ (Fig. 6e) showed two broad positive absorption bands at 400 nm and at 450–600 nm due to the formation of both the anionic radical of BQ and the cationic radical of PYZ *via* the sequential ET and HT after the photoexcitation of C-dots. A comparative kinetics study of C-dot–BQ, C-dot–PYZ, and C-dot–(PYZ + BQ) complexes at 400 nm (BQ radical) and 500 nm (PYZ radical) is shown in Fig. 6f and g. The fitting data show that the ET and HT times were 600 fs and 300 fs, respectively, from the photoexcited C-dots to the individual BQ and PYZ, but the ET and HT became faster (<100 fs) when the mixture of PYZ + BQ was present with C-dots. A model (Fig. 6b) is proposed for the ET and HT in the C-dot–(BQ + PYZ) system, along with the lifetime. Finally, significant degradation of methylene blue (MB) and rhodamine B (RhB) dyes was observed in the presence of both electron and hole transferring agents (PYZ + BQ) with C-dots. Thus, the combination of ET and HT agents enhances the PET process by promoting greater charge separation, suggesting that C-dots are promising metal-free photosensitizers for improving photocatalytic activity in the presence of pollutants.^77^

## 8. C-dot–hemin heterostructure

**Fig. 7:**
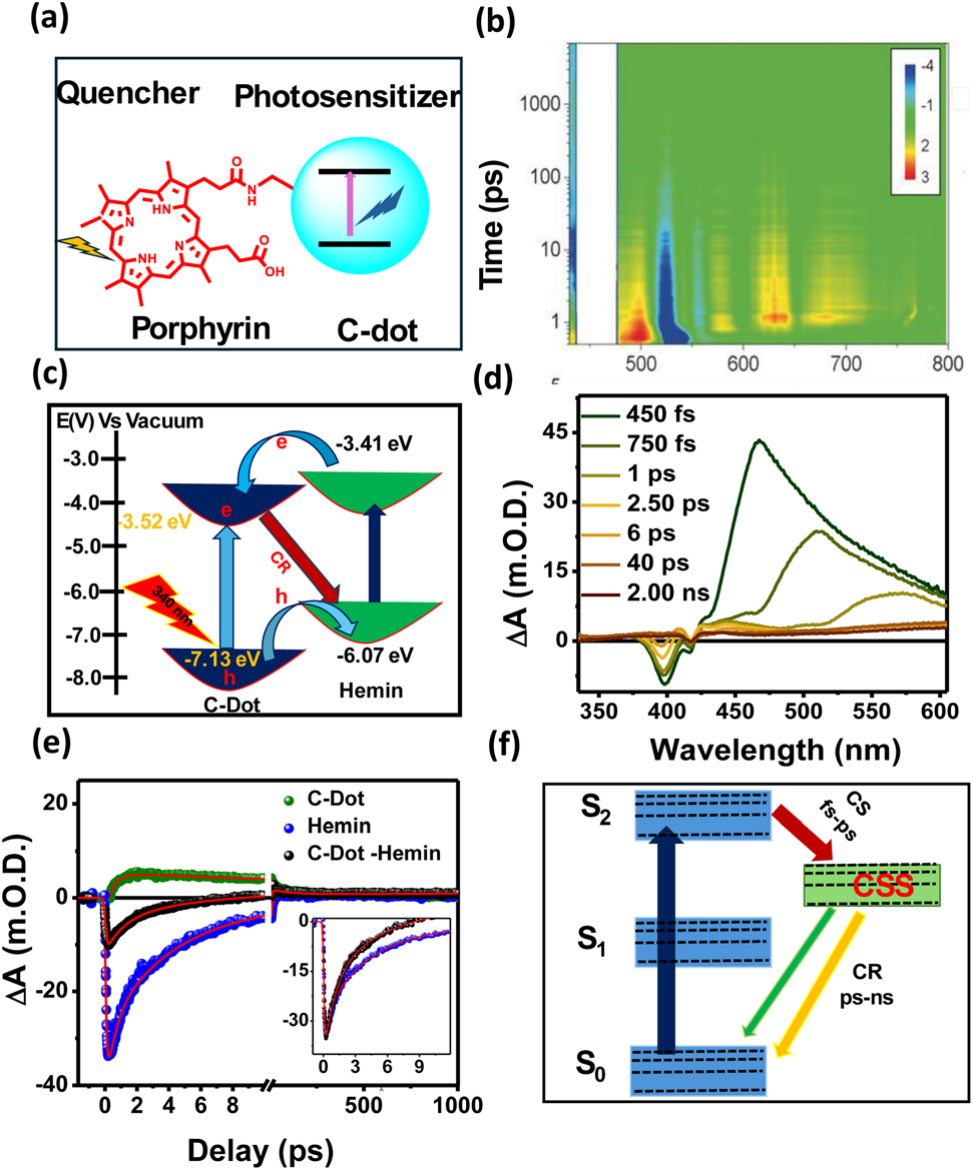
(a) Representation of porphyrin-conjugated C-dots. (b) Differential absorption 3D map obtained for fs-TA experiments on *meso*-tetraarylporphyrin-conjugated C-dots upon an excitation at 450 nm, adapted from ref. 72 with permission from Wiley, copyright 2025. (c) Different ET, HT, and CR pathways upon light excitations on C-dots and hemin. (d) TA spectra of C-dot–hemin HS at different delay times. (e) TA kinetics of bleach recovery at 400 nm of C-dot, hemin, and C-dot–hemin HS after exciting at 320 nm (insets: zoomed-in images of the first few ps of bleach recovery). (f) Different relaxation pathways upon selective excitation of hemin within the C-dot–hemin HS, adapted from ref. 94 with permission from the Royal Society of Chemistry, copyright 2025.

Heterostructures provide a means for creating new properties *via* sharing the electronic and optical properties of both materials. Here, we discuss C-dot-based heterostructures that are formed *via* covalently attached quenchers to the C-dots system, typically porphyrin-conjugated C-dots (Fig. 7a), to increase the charge separation process. In this context, the Guldi group conducted pioneering work on covalently porphyrin-conjugated C-dots and demonstrated that charge transfer occurs in both the ground and excited states, driven by strong electronic interactions present in the ground state. The differential absorption 3D map obtained from fs-TA experiments (Fig. 7b) on *meso*-tetraarylporphyrin-conjugated C-dots revealed four distinct transient components corresponding to the deactivation process.^72,93^ Further, we developed hemin (Fe-containing protoporphyrin)-conjugated C-dots *via* an EDC/NHS coupling reaction, where C-dots act as the photosensitizer and a tethered hemin molecule is the electron acceptor. Steady-state PL and time-resolved PL decay analyses revealed that rapid HT occurs from photoexcited C-dots to hemin upon C-dot excitation, while an ET process is observed upon hemin excitation, as shown in Fig. 7c. In the fs-TA spectrum (Fig. 7d), a broad positive absorbance peak observed at 400–550 nm signals the formation of the hemin radical species due to HT from the photoexcited C-dots to hemin. Comparative kinetics at 370 nm and 400 nm of free C-dots, hemin, and the C-dot–hemin HS showed an ultrafast HT process (<100 fs) in the C-dot–hemin HS (Fig. 7e). The ultrafast charge transfer process in C-dot–hemin HS makes them highly suitable for light-modulated applications. Most porphyrin-conjugated C-dots exhibit the formation of a charge-separated state on the femtosecond to picosecond timescale, while charge recombination occurs over tens of picoseconds to nanoseconds, as illustrated in Fig. 7f. Furthermore, the observed light-induced short-range charge transport serves as a basis for understanding long-range charge propagation within the biopolymeric matrix. When integrated into a protein-based biopolymeric matrix, the C-dot–hemin HS exhibited a charge transfer rate 3.3 times faster than in solution, indicating that the side-chain functional groups of the protein actively contribute to facilitating long-range, light-induced intermolecular charge separation.^94^ This hybrid material holds great promise for a wide range of optoelectronic research and applications.

## 9. C-dots as a photosensitizer in perovskite/C-dots composites

**Fig. 8:**
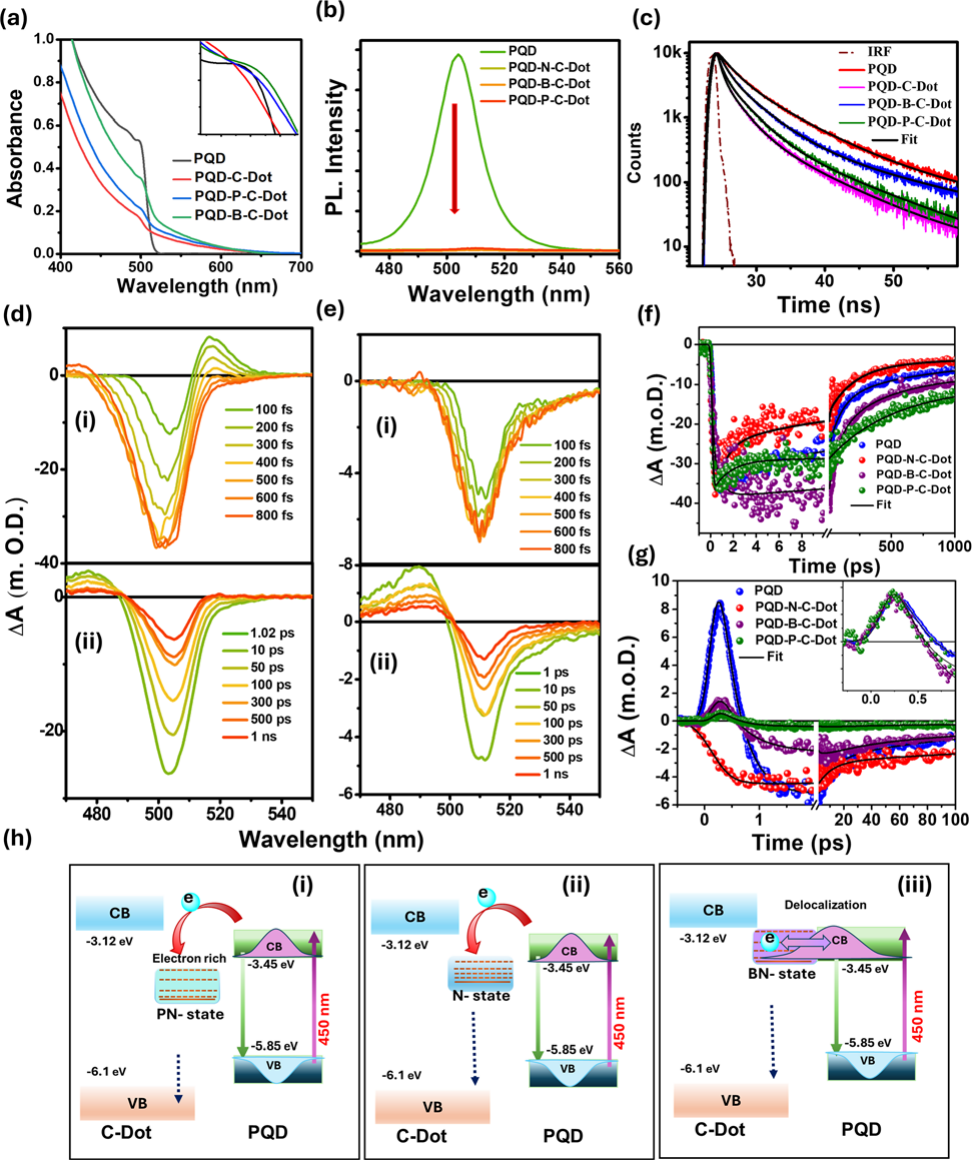
(a) Absorption spectra of PQD and PQD–C-dot composites. (b) Steady-state and (c) time-resolved PL spectra of the pure PQD and PQD–C-dot composite systems. (d) TA spectra of PQD at (i) early time scale (0–800 fs) and (ii) late time scale (1 ps to 1 ns); (e) TA spectra of PQD–N-C-dot composite system at (i) early time scale (0–800 fs) and (ii) late time scale (1 ps to 1 ns) after exciting at 420 nm. (f) Normalized bleaching kinetics (at 505 nm). (g) Biexciton kinetics (at 516 nm) of PQD and PQD–C-dot composite systems. (h) Different relaxation pathways of (i) PQD–P-N-C-dots, (ii) PQD–N-C-dots and (iii) PQD–B-N-C-dots. Adapted from ref. 76 with permission from the American Chemical Society, copyright 2025.

C-dots were utilized to fabricate perovskite/C-dot (PQD–C-dot) composites for dual purposes. In these systems, C-dots serve as carrier transport agents due to their excellent electron and hole accepting/donating capabilities, while also enhancing the photostability of CsPbBr_3_. Various PQD-doped C-dot composites (B- and P-co-doped nitrogen-rich C-dots, referred to as B-NC-dot and P-NC-dot) were synthesized using a simple heating method to investigate the influence of dopants on ultrafast carrier dynamics. The absorbance spectra (Fig. 8a) revealed a shift in the excitonic peak of PQDs (498 nm) in the composites, indicating excitonic interactions between the PQDs and doped C-dots. Additionally, a broad absorption band appeared in the 520–650 nm range after composite formation, suggesting the possibility of ground-state charge transfer from PQDs to C-dots. The PL quenching was observed in all PQD–C-dot composites (Fig. 8b), further confirming interaction between components. The average lifetime of PQDs (*τ* = 6.14 ns) was notably reduced in the composite systems: PQD–N-C-dots (〈*τ*〉 = 4.00 ns), PQD–B-C-dots (〈*τ*〉 = 5.21 ns), and PQD–P-C-dots (〈*τ*〉 = 5.32 ns), as shown in Fig. 8c. This reduction implies efficient photoexcited electron transfer from PQDs to the intermediate states in doped C-dots, likely due to favourable band alignment. The TA spectroscopic studies of PQD and PQD–C-dots were conducted to examine the carrier dynamics following 420 nm excitation, as shown in Fig. 8d(i), (ii), e(i) and (ii). The GSB at 505 nm and the biexciton peak at 526 nm both diminished significantly in intensity upon composite formation, indicating a reduced number of charge carriers in PQDs within the composites. The kinetic analysis at the bleaching position (Fig. 8f) demonstrated rapid electron transfer between photoexcited PQDs and both NC-dots and PC-dots. In PQD–B-C-dot composites, a delayed growth component was observed, suggesting hot carrier delocalization between PQDs and B-C-dots. Moreover, the biexciton decay in pristine PQDs (140 fs) became faster upon composite formation with B-C-dots and P-C-dots (110 fs for both), as shown in Fig. 7g. Kinetics details indicate that a new hybrid state was formed after doping the P and B with N-C-dots, as shown in Fig. 8h(i)–(iii). The fast dissociation of excitons and biexcitons enhances the photocatalytic efficiency of the PQD–C-dot composites.^76^

## 10. Summary and outlook

Over the past decade, we have aimed to establish a definitive understanding of the origin of fluorescence and excitation wavelength-dependent emission in various C-dots. To address this longstanding question, pure blue-, green-, and red-emitting C-dots were synthesized from identical starting materials and subsequently separated *via* column chromatography. In the first section, we identified the emissive centres using steady-state and time-resolved PL spectroscopy, along with ultrafast TA spectroscopy. Our findings revealed that the core state of C-dots is responsible for blue emission across all types of C-dots, which is an intrinsic feature. In contrast, variations in oxygen-containing surface functional groups create distinct surface states, which account for the green and red emissions. The second section of the perspective highlighted the potential of C-dots as photosensitizers in confined environments, doped systems, C-dot/molecular hybrids, and C-dot/perovskite composites. Through ultrafast TA spectroscopy, we explored carrier dynamics in both core and surface states. A key discovery was that ET can be modulated by selectively exciting core or surface states, owing to their heterogeneous nature. Notably, the core state exhibited superior photosensitization capabilities compared to surface states. Ultrafast carrier dynamics revealed pathways of core- and surface-assisted recombination, as well as ET and HT *via* both core- and surface-mediated mechanisms. We observed ultrafast ET and HT processes from photoexcited C-dots to various quenchers through adiabatic and non-adiabatic processes, effectively extending charge separation in C-dot-quencher systems. A summary of C-dot-based photosensitizers, along with their controlling factors, responsible charge carriers, and charge recombination times, is presented in Table 2. Finally, we will discuss the photosensitization potential of C-dots across different systems and outline key challenges and critical aspects for future research directions.

**Table 2:** Summary of the photosensitizer capability of C-dots, indicating the controlling factors, carriers responsible, and charge recombination time (*τ*_CR_)

Photosensitizer	Controlling factors	Carriers responsible	*τ*_CR_ (ns)	Ref.
C-dots	Micelle: (i) CTAB + DMA	Electron	2.10	75
	(ii) TTAB + DMA		2.20	
	(iii) DTAB + DMA		2.81	
C-dots	Revers micelle (AOT): (i) AOT + DMA	Electron	2.66	62
	(ii) AOT + DMA + MV^2+^	Electron + hole	2.22	
C-dots	Doping: N-doped	Electron	1.61	27
	B-doped		0.69	
	P-doped		0.68	
C-dots	Small molecule: (i) benzoquinone (BQ)	Electron	4.65	77
	(ii) Pyrazine (PYZ)	Hole	3.32	
	(iii) BQ + PYZ	Electron + hole	2.50	
C-dots	Covalent bond: (i) hemin/porphyrin	Hole	3.95	94
Perovskites	Heterostructure: N-C-dots	Electron	4.00	76
	B-C-dots	Electron	5.21	
	P-C-dots	Electron delocalization	5.32	

• Quantitative modelling of exciton migration: a combination of advanced spectroscopic and theoretical approaches is essential for modeling exciton migration. To fully understand the optical response to exciton migration, a real-time simulation that accounts for all light–matter, electron–electron, and electron–phonon interactions is required. Such efforts will contribute to improving the efficiency of C-dot-based photocatalysis, photosensing, photosensitization, optoelectronic, and bioelectronic devices.

• Structure–property correlations using *in situ* spectroscopy: due to the complex structure of C-dots, *in situ* spectroscopic investigations provide a powerful means to establish a comprehensive correlation between their optical properties and structural features, which is crucial for a complete understanding of their structure and for advancing a wide range of applications.

• Surface-modification strategy: the photosensitiser capability as either an electron donor or acceptor can be tuned by modification of the surface functional groups and surface charge of C-dots *via* the proper choice of surface capping agents or click chemistry.

• Manipulation of the core state: the photosensitiser capability of the core state is greater than that of the surface state of C-dots. Thus, control over the nitrogen contained in the core state can influence the photosensitiser capability of C-dots.

• Incorporation of chirality: chirality is one of the factors that can influence the carrier dynamics and photosensitiser capability of C-dots, which is not fully explored and needs further investigation for the development of chiral photosensitisers.

• AI-assisted synthesis for next-generation C-dot-based photosensitizers: the successful implementation of machine learning algorithms can aid in predicting, optimizing, and accelerating the synthesis, optical properties, and redox levels of C-dots, thereby facilitating the development of next-generation photosensitizers.

## Conflicts of interest

The authors declare no competing financial interest.

## Data Availability

It is hereby declared that no primary research results, software, or code have been included, and no new data were generated or analysed in the preparation of this review article.
